# Supplementary material for: From sequence to enzyme mechanism using multi-label machine learning
Source: BMC Bioinformatics. 2014 May 19;15:150. doi: 10.1186/1471-2105-15-150 (PMC4229970; doi:10.1186/1471-2105-15-150)
Supplement: Additional file 2 — Java code of ml2db. Additional file ml2db_code.tar.gz contains the Java source code to run the multi-label machine learning experiments and save the results to database. The code’s Javadoc is included. [file 1471-2105-15-150-S2.zip › additional file 2/ml2db/ml2db/doc/test/dataharness/TestProjectParameters.html]

TestProjectParameters


---


|  |  |  |
| --- | --- | --- |
| **Overview** \| **Package** \| **Class** \| **Use** \| **Tree** \| **Deprecated** \| **Index** \| **Help** | |  |
| **PREV CLASS**   **NEXT CLASS** | **FRAMES**    **NO FRAMES**     **All Classes** |  |
| SUMMARY: NESTED \| FIELD \| CONSTR \| METHOD | DETAIL: FIELD \| CONSTR \| METHOD |  |


---


## test.dataharness Class TestProjectParameters

```
java.lang.Object
  uk.ac.ed.inf.enzml.ProjectParameters
      test.dataharness.TestProjectParameters
```

---

``` public class TestProjectParameters extends ProjectParameters ```

---

| **Field Summary** | |
| --- | --- |
| `static boolean` | `DEBUG` |
| `static java.lang.String` | `FILTER_OLD_TEST_SET` |
| `static java.lang.String` | `FILTER_TRAIN_SET` |
| `static java.lang.String` | `INSTANCE_1_2` |
| `static java.lang.String` | `INSTANCE_1_2_EMPTY` |
| `static java.lang.String` | `INSTANCE_3` |
| `static java.lang.String` | `TEST_ARFF_DB_PROPS` |
| `static java.lang.String` | `TEST_ARFF_FILES_PATH` |
| `static java.lang.String` | `TEST_ARFF_PROPS_1` |
| `static java.lang.String` | `TEST_ARFF_PROPS_2` |
| `static java.lang.String` | `TEST_ARFF_PROPS_NO_XML` |
| `static java.lang.String` | `TEST_ARFF_PROPS_PATH` |
| `static java.lang.String` | `TEST_BASE_DATA_PATH` |
| `static java.lang.String` | `TEST_BASE_PATH` |
| `static java.lang.String` | `TEST_DATA_DIR` |
| `static java.lang.String` | `TEST_DB_CONN_PROPERTIES` |
| `static java.lang.String` | `TEST_FIGURES_PATH` |
| `static java.lang.String` | `TEST_MACHINE_LEARNING_PROPS` |
| `static java.lang.String` | `TEST_ML_PROPS_PATH` |
| `static java.lang.String` | `TEST_MODEL_FOLDER` |
| `static java.lang.String` | `TEST_PLOTS_FOLDER` |
| `static java.lang.String` | `TEST_PREDICTIONS_FOLDER` |
| `static java.lang.String` | `TEST_PROPS_PATH` |
| `static java.lang.String` | `TEST_RESULTS_DIRECTORY_ACTIVE_LEARNING` |
| `static java.lang.String` | `TEST_RESULTS_FOLDER` |

**Fields inherited from class uk.ac.ed.inf.enzml.ProjectParameters**

`ARFF_PROPS_APHIDICOLA, ARFF_PROPS_ARCHAEA, ARFF_PROPS_BACTERIA, ARFF_PROPS_ECOLI, ARFF_PROPS_ECOLI_K12ALL, ARFF_PROPS_EUKARIA, ARFF_PROPS_FALCIPARUM, ARFF_PROPS_FUNGI, ARFF_PROPS_GENITALIUM, ARFF_PROPS_HUMAN, ARFF_PROPS_INFLUENZAE, ARFF_PROPS_INVERTEBRATES, ARFF_PROPS_NON_APHIDICOLA, ARFF_PROPS_NON_ECOLI, ARFF_PROPS_NON_ECOLIK12ALL, ARFF_PROPS_NON_FALCIPARUM, ARFF_PROPS_NON_GENITALIUM, ARFF_PROPS_NON_INFLUENZAE, ARFF_PROPS_NON_PNEUMONIAE, ARFF_PROPS_NOT_HUMAN, ARFF_PROPS_PATH, ARFF_PROPS_PLANTS, ARFF_PROPS_PNEUMONIAE, ARFF_PROPS_RANDOM_100, ARFF_PROPS_RANDOM_ARCHAEA, ARFF_PROPS_RANDOM_BACTERIA, ARFF_PROPS_RANDOM_EUKARIA, ARFF_PROPS_RANDOM_FUNGI, ARFF_PROPS_RANDOM_INVERTEBRATES, ARFF_PROPS_RANDOM_PLANTS, ARFF_PROPS_RANDOM_VERTEBRATES, ARFF_PROPS_SWISSKEGG, ARFF_PROPS_TK_NOTIN_SKUNIREF50, ARFF_PROPS_TREMBL_KEGG, ARFF_PROPS_TREMBL_KEGG_EC1, ARFF_PROPS_TREMBL_KEGG_EC2, ARFF_PROPS_TREMBL_KEGG_EC3, ARFF_PROPS_TREMBL_KEGG_EC4, ARFF_PROPS_TREMBL_KEGG_EC5, ARFF_PROPS_TREMBL_KEGG_EC6, ARFF_PROPS_TREMBL_KEGG_NOEC, ARFF_PROPS_VERTEBRATES, ARFF_TABLE, AUTHOR, DATA_LOCAL_DB, DATA_REMOTE_DB, EXPECTED_NUMBER_OF_UNIQUE_ATTRIBUTES, EXPECTED_NUMBER_OF_UNIQUE_INSTANCES, FIGURES_PATH, MACHINE_LEARNING_LOCAL_DB_PROPS, MACHINE_LEARNING_PROPS, MACHINE_LEARNING_REMOTE_DB_PROPS, MODELS_PATH, PREDICTIONS_PATH, PROJECT_NAME, RESULTS_DIRECTORY, RESULTS_DIRECTORY_ACTIVE_LEARNING, SOFTWARE_PROJECT_NAME, STATISTICS_PATH, VERSION, WEB_PAGE`


**Constructor Summary**

`TestProjectParameters()`


| **Method Summary** | |
| --- | --- |
| `static DbManagers` | `getArffRealRestTestDbManagers()` |
| `static DbManagers` | `getDbManagers()` |
| `static uk.ac.ed.inf.utils.database.DbManager` | `getTestArffRecordsDbManager()` |
| `static uk.ac.ed.inf.utils.database.DbManager` | `getTestDataDbManager()` |
| `static uk.ac.ed.inf.utils.database.DbManager` | `getTestMachineLearningResultsDbManager()` |

**Methods inherited from class uk.ac.ed.inf.enzml.ProjectParameters**

`getLocalDbManagers, getRemoteDbManagers`

**Methods inherited from class java.lang.Object**

`equals, getClass, hashCode, notify, notifyAll, toString, wait, wait, wait`

**Field Detail**

### DEBUG

```
public static final boolean DEBUG
```

**See Also:**: Constant Field Values

---


### TEST\_BASE\_PATH

```
public static final java.lang.String TEST_BASE_PATH
```

**See Also:**: Constant Field Values

---


### TEST\_DATA\_DIR

```
public static final java.lang.String TEST_DATA_DIR
```

**See Also:**: Constant Field Values

---


### TEST\_BASE\_DATA\_PATH

```
public static final java.lang.String TEST_BASE_DATA_PATH
```

**See Also:**: Constant Field Values

---


### TEST\_PREDICTIONS\_FOLDER

```
public static final java.lang.String TEST_PREDICTIONS_FOLDER
```

**See Also:**: Constant Field Values

---


### TEST\_PLOTS\_FOLDER

```
public static final java.lang.String TEST_PLOTS_FOLDER
```

**See Also:**: Constant Field Values

---


### TEST\_PROPS\_PATH

```
public static final java.lang.String TEST_PROPS_PATH
```

**See Also:**: Constant Field Values

---


### TEST\_ARFF\_FILES\_PATH

```
public static final java.lang.String TEST_ARFF_FILES_PATH
```

**See Also:**: Constant Field Values

---


### TEST\_ARFF\_PROPS\_PATH

```
public static final java.lang.String TEST_ARFF_PROPS_PATH
```

**See Also:**: Constant Field Values

---


### TEST\_ML\_PROPS\_PATH

```
public static final java.lang.String TEST_ML_PROPS_PATH
```

**See Also:**: Constant Field Values

---


### TEST\_ARFF\_PROPS\_NO\_XML

```
public static final java.lang.String TEST_ARFF_PROPS_NO_XML
```

**See Also:**: Constant Field Values

---


### TEST\_MACHINE\_LEARNING\_PROPS

```
public static final java.lang.String TEST_MACHINE_LEARNING_PROPS
```

**See Also:**: Constant Field Values

---


### TEST\_ARFF\_PROPS\_1

```
public static final java.lang.String TEST_ARFF_PROPS_1
```

**See Also:**: Constant Field Values

---


### TEST\_ARFF\_PROPS\_2

```
public static final java.lang.String TEST_ARFF_PROPS_2
```

**See Also:**: Constant Field Values

---


### TEST\_ARFF\_DB\_PROPS

```
public static final java.lang.String TEST_ARFF_DB_PROPS
```

**See Also:**: Constant Field Values

---


### TEST\_DB\_CONN\_PROPERTIES

```
public static final java.lang.String TEST_DB_CONN_PROPERTIES
```

**See Also:**: Constant Field Values

---


### TEST\_RESULTS\_FOLDER

```
public static final java.lang.String TEST_RESULTS_FOLDER
```

**See Also:**: Constant Field Values

---


### TEST\_RESULTS\_DIRECTORY\_ACTIVE\_LEARNING

```
public static final java.lang.String TEST_RESULTS_DIRECTORY_ACTIVE_LEARNING
```

**See Also:**: Constant Field Values

---


### TEST\_MODEL\_FOLDER

```
public static final java.lang.String TEST_MODEL_FOLDER
```

**See Also:**: Constant Field Values

---


### FILTER\_OLD\_TEST\_SET

```
public static final java.lang.String FILTER_OLD_TEST_SET
```

**See Also:**: Constant Field Values

---


### FILTER\_TRAIN\_SET

```
public static final java.lang.String FILTER_TRAIN_SET
```

**See Also:**: Constant Field Values

---


### INSTANCE\_1\_2

```
public static final java.lang.String INSTANCE_1_2
```

**See Also:**: Constant Field Values

---


### INSTANCE\_1\_2\_EMPTY

```
public static final java.lang.String INSTANCE_1_2_EMPTY
```

**See Also:**: Constant Field Values

---


### INSTANCE\_3

```
public static final java.lang.String INSTANCE_3
```

**See Also:**: Constant Field Values

---


### TEST\_FIGURES\_PATH

```
public static final java.lang.String TEST_FIGURES_PATH
```

**See Also:**: Constant Field Values


**Constructor Detail**

### TestProjectParameters

```
public TestProjectParameters()
```


**Method Detail**

### getArffRealRestTestDbManagers

```
public static DbManagers getArffRealRestTestDbManagers()
```

:   **Returns:**

---


### getDbManagers

```
public static DbManagers getDbManagers()
```

---


### getTestArffRecordsDbManager

```
public static uk.ac.ed.inf.utils.database.DbManager getTestArffRecordsDbManager()
```

---


### getTestDataDbManager

```
public static uk.ac.ed.inf.utils.database.DbManager getTestDataDbManager()
```

---


### getTestMachineLearningResultsDbManager

```
public static uk.ac.ed.inf.utils.database.DbManager getTestMachineLearningResultsDbManager()
```


---


|  |  |  |
| --- | --- | --- |
| **Overview** \| **Package** \| **Class** \| **Use** \| **Tree** \| **Deprecated** \| **Index** \| **Help** | |  |
| **PREV CLASS**   **NEXT CLASS** | **FRAMES**    **NO FRAMES**     **All Classes** |  |
| SUMMARY: NESTED \| FIELD \| CONSTR \| METHOD | DETAIL: FIELD \| CONSTR \| METHOD |  |


---
